# Intermediate Conductance Ca^2+^-Activated K^+^ Channels Modulate Human Placental Trophoblast Syncytialization

**DOI:** 10.1371/journal.pone.0090961

**Published:** 2014-03-03

**Authors:** Paula Díaz, Amber M. Wood, Colin P. Sibley, Susan L. Greenwood

**Affiliations:** 1 Maternal and Fetal Health Research Centre, Institute of Human Development, Faculty of Medical and Human Sciences, University of Manchester, Manchester, United Kingdom; 2 St. Mary’s Hospital, Central Manchester University Hospitals National Health Service (NHS) Foundation Trust, Manchester Academic Health Science Centre, Manchester, United Kingdom; Xavier Bichat Medical School, INSERM-CNRS - Université Paris Diderot, France

## Abstract

Regulation of human placental syncytiotrophoblast renewal by cytotrophoblast migration, aggregation/fusion and differentiation is essential for successful pregnancy. In several tissues, these events are regulated by intermediate conductance Ca^2+^-activated K^+^ channels (IK_Ca_), in part through their ability to regulate cell volume. We used cytotrophoblasts in primary culture to test the hypotheses that IK_Ca_ participate in the formation of multinucleated syncytiotrophoblast and in syncytiotrophoblast volume homeostasis. Cytotrophoblasts were isolated from normal term placentas and cultured for 66 h. This preparation recreates syncytiotrophoblast formation *in vivo*, as mononucleate cells (15 h) fuse into multinucleate syncytia (66 h) concomitant with elevated secretion of human chorionic gonadotropin (hCG). Cells were treated with the IK_Ca_ inhibitor TRAM-34 (10 µM) or activator DCEBIO (100 µM). Culture medium was collected to measure hCG secretion and cells fixed for immunofluorescence with anti-IK_Ca_ and anti-desmoplakin antibodies to assess IK_Ca_ expression and multinucleation respectively. K^+^ channel activity was assessed by measuring ^86^Rb efflux at 66 h. IK_Ca_ immunostaining was evident in nucleus, cytoplasm and surface of mono- and multinucleate cells. DCEBIO increased ^86^Rb efflux 8.3-fold above control and this was inhibited by TRAM-34 (85%; *p*<0.0001). Cytotrophoblast multinucleation increased 12-fold (*p*<0.05) and hCG secretion 20-fold (*p*<0.05), between 15 and 66 h. Compared to controls, DCEBIO reduced multinucleation by 42% (*p*<0.05) and hCG secretion by 80% (*p*<0.05). TRAM-34 alone did not affect cytotrophoblast multinucleation or hCG secretion. Hyposmotic solution increased ^86^Rb efflux 3.8-fold (*p*<0.0001). This effect was dependent on extracellular Ca^2+^, inhibited by TRAM-34 and 100 nM charybdotoxin (85% (*p*<0.0001) and 43% respectively) but unaffected by 100 nM apamin. In conclusion, IK_Ca_ are expressed in cytotrophoblasts and their activation inhibits the formation of multinucleated cells *in vitro*. IK_Ca_ are stimulated by syncytiotrophoblast swelling implicating a role in syncytiotrophoblast volume homeostasis. Inappropriate activation of IK_Ca_ in pathophysiological conditions could compromise syncytiotrophoblast turnover and volume homeostasis in pregnancy disease.

## Introduction

The syncytiotrophoblast is the transporting epithelium of the human placenta being the interface between maternal and fetal blood. This highly specialized epithelial cell also performs a number of other functions including hormone production and secretion. Syncytiotrophoblast has a short life span and is renewed by cellular turnover in a tightly regulated process where proliferative mononucleate cytotrophoblasts exit the cell cycle, differentiate and fuse with the overlying syncytial layer [1]; both apoptosis and autophagy have been hypothesized to play a role in completing turnover [2], [3].

*In vitro* models have been used to study some of the features of syncytiotrophoblast turnover. These include cytotrophoblasts isolated from normal term placenta and maintained in primary culture [4], [5]. After 15–18 h of culture, cytotrophoblasts are predominantly mononucleate and secrete small amounts of human chorionic gonadotropin (hCG). Over 24–66 h they migrate, aggregate and fuse to become multinucleated, a process reminiscent of syncytiotrophoblast formation *in vivo* [5]–[7]. This morphological differentiation is associated with a several-fold increase in the production and secretion of hCG. hCG, which is synthesized and secreted by terminally differentiated syncytiotrophoblast [8], is one key regulator of cytotrophoblast biology and acts in an autocrine/paracrine manner to facilitate syncytiotrophoblast renewal by promoting cytotrophoblast differentiation and fusion [9].

The importance of syncytiotrophoblast renewal for the progression of normal pregnancy is highlighted by the fact that its dysregulation is linked to pregnancy complications associated with maternal and/or fetal morbidity and mortality, in particular pre-eclampsia [10]–[12], fetal growth restriction [11]–[13] and maternal obesity [14]. In pre-eclampsia there is elevated cytotrophoblast proliferation [11], [15], [16] and apoptosis [11], [17]–[19], and a greater number of syncytial nuclear aggregates [20], compared to normal pregnancy. Furthermore, there is evidence to suggest that a rate-limiting step for syncytiotrophoblast formation, cytotrophoblast fusion, is reduced in pre-eclampsia [12], [21]. Cytotrophoblasts isolated from placentas of women with pre-eclampsia have a lower rate of syncytialization than those of normal pregnancy [22]. Expression of syncytin-1 [23] and syncytin-2 [24], envelope fusogenic proteins that induce syncytium formation [23], [25], [26] is downregulated both in isolated cytotrophoblasts and placental villous tissue from pregnancies complicated with pre-eclampsia [22], [24], [27], [28]. Syncytiotrophoblast expression of other fusogenic proteins, for example e-cadherin [16], is also reduced in pre-eclampsia. Collectively, dysregulation of the processes contributing to syncytiotrophoblast renewal culminates in a decrease in the total volume of syncytiotrophoblast in pregnancies complicated by pre-eclampsia and fetal growth restriction [29]. This has implications for nutrient delivery to the fetus as syncytiotrophoblast volume correlates with fetal weight [30]. However, the intracellular and extracellular signals that trigger and regulate cytotrophoblast fusion to form syncytiotrophoblast are not well understood.

In non-placental tissues, cellular proliferation, fusion and apoptosis can be regulated by members of the Ca^2+^-activated K^+^ channel (K_Ca_) family, in particular by intermediate conductance Ca^2+^-activated K^+^ channels (IK_Ca_; K_Ca_3.1; single channel conductance 50–200 pS). IK_Ca_s are voltage-insensitive and are strongly activated by increased concentrations of intracellular Ca^2+^ ([Ca^2+^]_i_; 300–700 nM) [31], [32]. IK_Ca_ mRNA was shown to be highly expressed by human placenta over 15 years ago [33] but the functions of IK_Ca_ in the placenta have not been explored.

A major function of IK_Ca_ is to regulate cellular volume [34]–[38]. IK_Ca_ activation induces K^+^ efflux from cells, which both lowers intracellular K^+^ concentration and promotes the loss of water by osmosis to induce cell shrinkage [39]. Appropriate adjustment of cell volume and/or intracellular K^+^ concentration is essential for cells to undergo proliferation, migration, fusion and apoptosis [40]. Indeed, in non-placental tissues, IK_Ca_ has been shown to contribute to tissue homeostasis by regulating proliferation [31], [41]–[43], differentiation/fusion [44], [45], cell migration [46]–[48] and apoptosis [49]. The ability of IK_Ca_ to regulate cell volume has been revealed experimentally by exposing cells to an osmotic challenge [34], [35], [37], [50]. When placed in hyposmotic solutions, cells initially swell but then restore their volume by a process of regulatory volume decrease (RVD). In many cells hyposmotic cell swelling elevates intracellular Ca^2+^ which activates IK_Ca_, promotes K^+^ efflux and water follows to achieve RVD [34]. However, a role for IK_Ca_ in regulating renewal of syncytiotrophoblast and/or syncytiotrophoblast volume has yet to be explored.

We tested the hypotheses that IK_Ca_ participates in the formation of multinucleate syncytiotrophoblast and that IK_Ca_ has a role in syncytiotrophoblast volume regulation. Using isolated cytotrophoblasts in primary culture we confirmed IK_Ca_ protein expression and tested the effects of IK_Ca_ modulators on ^86^Rb efflux, the formation of multinucleate syncytia and the secretion of hCG. To investigate whether IK_Ca_ participate in syncytiotrophoblast RVD, cells were exposed to hyposmotic solutions and ^86^Rb efflux measured in the presence and absence of IK_Ca_ modulators.

## Materials and Methods

### Materials

Unless otherwise stated, all chemicals were from Sigma-Aldrich (Poole, UK).

### Ethics Statement

Human placentas used in this study were obtained from St. Mary’s Hospital Maternity Unit (Manchester, UK) following written informed consent as approved by the Local Research Ethics Committee (North West (Haydock Park) Research Ethics Committee (Ref: 08/H1010/55)). Placentas were collected at term (37–42 weeks) following uncomplicated pregnancy and delivery of a healthy baby by vaginal or Caesarean section. Exclusion criteria were body mass index >30 (measured at booking), pregnancy hypertension/pre-eclampsia, fetal growth restriction, gestational diabetes. The investigation conforms to the principles outlined in the Declaration of Helsinki.

### Cytotrophoblast Isolation

Cytotrophoblasts were isolated from normal term placentas and cultured for 66 h. This is a well-characterized method [4], [5], [51]–[54] which recreates syncytiotrophoblast formation *in vivo*, as mononucleate cells (15 h) fuse into multinucleate syncytia (66 h) concomitant with elevated secretion of hCG.

Cytotrophoblasts were obtained using an adaptation of the method used by Kliman *et al*. [5], as previously described [4]. Briefly, full thickness placenta samples (∼2 cm^3^) were taken within 30 min of delivery and placed into sterile saline. Placental villous tissue was further dissected from each sample after removal of the chorionic plate and decidua. ∼30 g of villous tissue were obtained and submitted to digestion 3 times in Hank’s balanced salt solution containing 2.5% trypsin and 0.2 mg/ml deoxyribonuclease (DNAse I) for 30 min at 37°C (in agitation). After each digestion, 100 ml of supernatant were obtained, layered onto 5 ml newborn calf serum and spun for 10 min at 2200 rpm (1000×*g*) at 20°C. Afterwards, pellets were resuspended in 1 ml Dulbecco’s modified Earle’s medium (DMEM; Invitrogen, Paisley, UK) and centrifuged for 10 min at 2200 rpm. The supernatant was discarded and the pellet resuspended in 6 ml DMEM and layered onto a discontinuous Percoll density gradient and centrifuged for 30 min at 2800 rpm (1500×*g*). The bands between 35–55% Percoll were obtained and mixed with cell culture medium (DMEM: Ham’s F-12 Nutrient Mixture (Invitrogen, Paisley, UK) 1∶1, 10% fetal calf serum (heat inactivated), 1% gentamicin, 0.2% benzylpenicillin, 0.2% streptomycin, 0.6% glutamine), before centrifugation at 2200 rpm for 10 min. The final pellet was resuspended in 2 ml of cell culture medium. Cells were plated onto 35 mm culture dishes (Nunc, Fisher Scientific, Loughborough, UK) in cell culture medium, or 16 mm coverslips in 12-well culture plates, at densities of 1–1.3×10^6^/ml and 1×10^6^/ml respectively at 37°C in a humidified incubator (95% air/5% CO_2_).

### Cytotrophoblast Primary Culture and Treatment

Cytotrophoblasts plated onto 16 mm coverslips were cultured for 66 h. Cultures were washed 3 times with phosphate-buffered saline (PBS) and cell culture medium was replaced with fresh medium at 15 and 42 h. Cells were untreated (control) or treated at 3, 15 and 42 h with IK_Ca_ modulators 100 µM DCEBIO (5, 6-dichloro-1-ethyl-1, 3-dihydro-2H-benzimidazol-2-one; IK_Ca_ activator) or 10 µM TRAM-34 (1-[(2-chlorophenyl) diphenylmethyl]-1H-pyrazole; IK_Ca_ inhibitor). In both cases, the final concentration of dimethyl sulfoxide (DMSO) in the cell culture medium was 0.1%. Previous studies from this laboratory have shown that DMSO at 0.1% does not alter cytotrophoblast morphological or biochemical differentiation [51].

At 15, 42 and 66 h of culture, cell culture medium was collected and stored at −20°C for measurement of β-hCG (hCG β-subunit; produced by terminally differentiated syncytiotrophoblast, used to assess cytotrophoblast biochemical differentiation [51]). Coverslips were placed into 1 ml 0.3 M NaOH, cells scraped and the cell lysate stored at 4°C. These samples were used to measure protein content (mg) with Bio-Rad Protein Assay, based on the Bradford method (Bio-Rad Laboratories, Hempstead, UK).

In addition, at 15, 42 and 66 h of culture, cells were fixed in absolute methanol (permeabilizing fixative; to detect intracellular immunostaining) for 20 min at −20°C or in 4% paraformaldehyde (PFA; non-permeabilizing fixative; to detect immunostaining associated with cellular surface) for 15 min at room temperature and stored in PBS at 4°C prior to immunofluorescence staining.

### Measurement of Cytotrophoblast hCG Secretion

The β-subunit of hCG is secreted by terminally differentiated syncytiotrophoblast and was used as an indicator of cytotrophoblast differentiation in culture [51]. β-hCG was assayed in cell-conditioned culture medium at 15, 42 and 66 h of culture by ELISA (DRG Diagnostics, Marburg, Germany). Thawed samples were used following the instructions of the manufacturer. Optical density was measured at 450 nm using a VersaMax microplate reader (Molecular Devices, CA, USA). hCG secretion was expressed as mIU/ml/mg protein.

### Immunofluorescent Staining

Methanol and PFA-fixed cells on 16 mm coverslips were washed in tris-buffered saline (TBS). Block of non-specific binding was performed for 30 min with 4% bovine serum albumin (BSA) in TBS. Cells were incubated for 1 h at room temperature with mouse monoclonal antibody to desmoplakin I+II (clone 2Q400; Abcam, Cambridge, UK), diluted 1∶100 in TBS or mouse monoclonal antibody to IK_Ca_ (K_Ca_3.1; clone 6C1; extracellular epitope; Alomone labs, Jerusalem, Israel), diluted 1∶50 in 1% BSA in TBS. Negative control was obtained by omission of the primary antibody. Cells were washed with TBS and the secondary antibody, FITC-polyclonal rabbit anti-mouse immunoglobulin (Dako, Cambridgeshire, UK) diluted 1∶50 in TBS, was applied and cells incubated for 1 h at room temperature in the dark. After washing with TBS, coverslips were mounted using Vectashield mounting medium with propidium iodide nuclear counterstain (PI; Vector labs, Peterborough, UK). Immunofluorescent images were captured using a Zeiss AxioObserver Inverted Microscope (magnification 400×).

### Analysis of Cytotrophoblast Multinucleation

Microscope images of cytotrophoblasts stained for desmoplakin and nuclei were used to assess multinucleation as a measurement of cytotrophoblast morphological differentiation. Based on a previously published method [51], [55], 2–3 observers counted the total number of nuclei per given field and the number of nuclei in syncytium (multinucleated cell defined as ≥3 nuclei within desmoplakin boundaries) using ImageJ 1.45 software (National Institutes of Health, USA). The number of multinucleated cells was expressed as a percentage of the total number of nuclei within a given field (% of nuclei in multinucleate cells).

### ^86^Rb Efflux from Cytotrophoblasts

^86^Rb is commonly used as a tracer of K^+^ and it has been previously shown that K_Ca_s are permeable to ^86^Rb [33]. ^86^Rb efflux was measured in cytotrophoblasts at 66 h of culture using a technique previously described [53]. Briefly, cells plated onto 35 mm dishes were removed from the incubator and washed in control Tyrode’s buffer (135 mM NaCl, 5 mM KCl, 1.8 mM CaCl_2_, 1 mM MgCl_2_, 10 mM HEPES, 5.6 mM glucose, pH 7.4; osmolality ∼283 mOsm/kgH_2_O, isotonic compared to maternal plasma at term [56]; osmolality measured by freezing point depression). Cells were incubated with 1 ml 4 µCi/ml ^86^Rb (89.7 µM; concentration 1 µCi/ml; stock activity 1 mCi) for 2 h at room temperature. After washing for 3 min in 2×25 ml Tyrode’s buffer (with no added isotope), ^86^Rb efflux was measured by the sequential addition and removal of 1 ml Tyrode’s buffer at 1 min intervals; samples were collected every 1 min over 15 min (control, basal ^86^Rb efflux) and/or exposed to various treatments over 5–15 min (experimental period): 10 µM TRAM-34, 100 µM DCEBIO, 100 nM apamin (small conductance Ca^2+^-activated K^+^ channel (SK_Ca_) inhibitor), 100 nM charybdotoxin (ChTx; IK_Ca_/large conductance Ca^2+^-activated K^+^ channel (BK_Ca_) inhibitor), hyposmotic solution (55 mM NaCl, 5 mM KCl, 1.8 mM CaCl_2_, 1 mM MgCl_2_, 10 mM HEPES, 5.6 mM glucose; pH 7.4; osmolality 145 mOsm/kgH_2_O), Ca^2+^-free hyposmotic solution (extracellular Ca^2+^ was buffered by removing CaCl_2_ and adding 0.5 mM EGTA). When used together, a pre-block/inhibition with TRAM-34 was performed at min 4 before adding DCEBIO-TRAM-34 or hyposmotic solution-TRAM-34. In a different set of experiments, ^86^Rb efflux was measured in efflux buffer with osmolality ranging 283–138 mOsm/kgH_2_O, which was obtained by varying the NaCl concentration.

After 15 min, the cells were lysed in 0.3 M NaOH for ∼1 h and scraped in order to release intracellular ^86^Rb which was then counted in the supernatant to give a measure of total ^86^Rb remaining in the cells at the end of the experiment (cellular ^86^Rb). Effluxed and cellular ^86^Rb was measured in a gamma-counter (Packard Cobra II Auto Gamma, CA, USA). All counts recorded were at least 10 times higher than background counts.

The time course of percentage (%) ^86^Rb efflux was calculated at each time point as ((^86^Rb effluxed/^86^Rb in cells) x100). The efflux rate constant was also determined making the assumption that ^86^Rb efflux at steady state reflects the loss of ^86^Rb from a single compartment (syncytiotrophoblast) limited by the K^+^ permeability of the plasma membrane. Consequently, the loss of ^86^Rb was measured by a first-order rate constant which was calculated over 10 min experimental period as (l_n_(^86^Rb in cell at time t/^86^Rb in cell at t_0_)) where t_0_ is the cellular ^86^Rb at the start of the experiment.

### Expression of Results and Statistics

Statistical analysis was performed using GraphPad Prism version 5 software. hCG secretion and multinucleation from control untreated cytotrophoblasts was expressed as mean ± standard error (SE) with n as the number of placentas. hCG secretion and multinucleation in TRAM-34 and DCEBIO-treated cells was expressed as median ± interquartile range (IQR) and analyzed with Friedman’s test with Dunn’s post hoc test. The relationship between ^86^Rb efflux and extracellular fluid osmolality was analyzed comparing control vs. each experimental osmolality using ANOVA with Turkey Kramer multicomparison post hoc test. Each value was expressed as mean ± SE. %^86^Rb efflux from multinucleated cytotrophoblasts was expressed as mean ± SE for each time point. The effects of all treatments on ^86^Rb efflux were assessed for statistical significance by comparing the differences in the slopes and intercepts of the rate constants using least squares linear regression analysis. In all cases, a *p* value less than 0.05 was considered statistically significant.

## Results

### Expression of IK_Ca_ in Cytotrophoblasts

**Figure 1:**
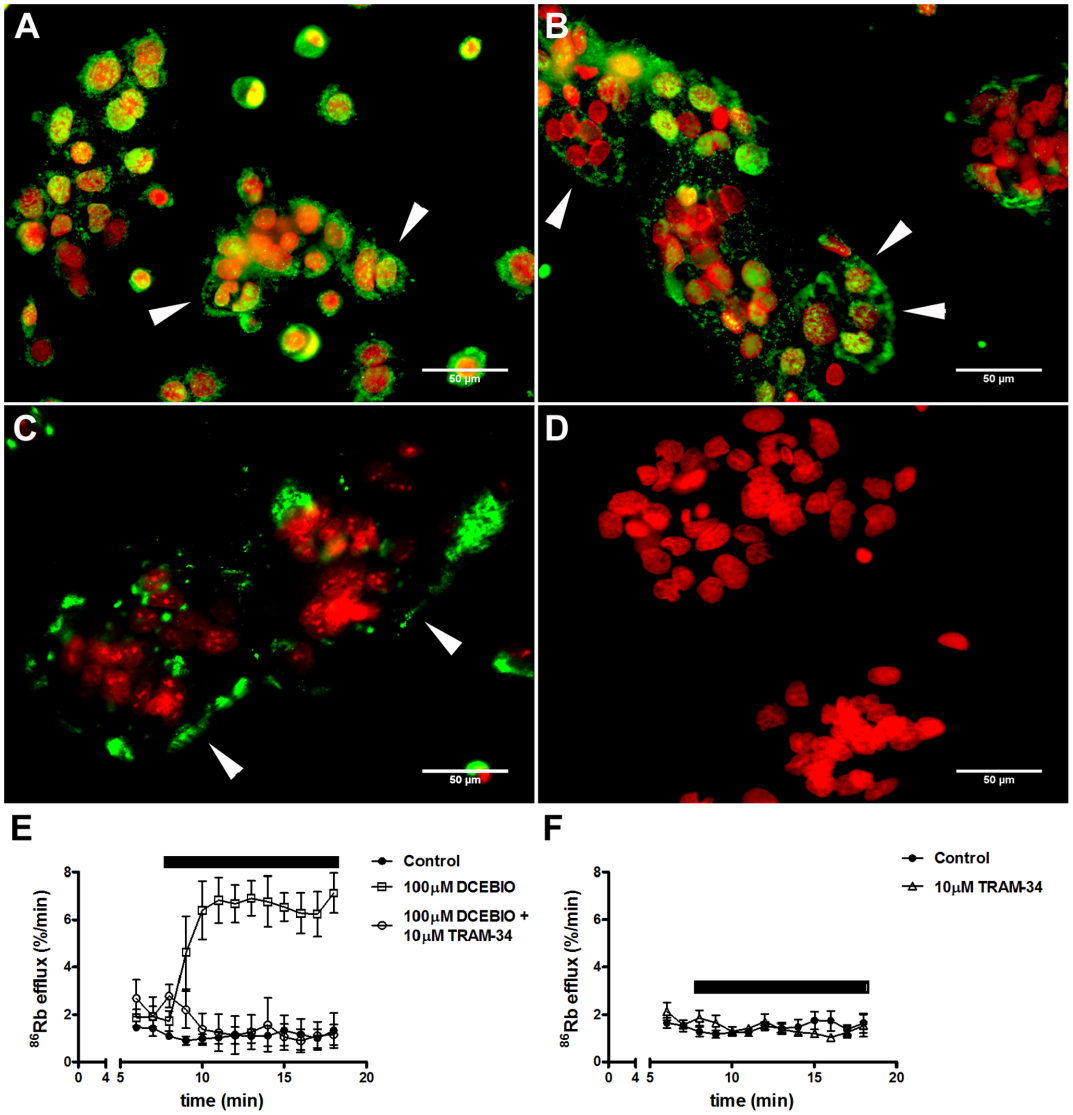
Expression of IK_Ca_ in placental cytotrophoblasts. IK_Ca_ protein expression is shown in representative images of dual immunofluorescent staining for IK_Ca_ (K_Ca_3.1; green) and nuclear counterstain (red) in cytotrophoblasts at 15 h (***A***) and 66 h (***B***, methanol-fixed cells; ***C***, PFA-fixed cells) of culture. Arrows indicate IK_Ca_ staining associated with cell surface. ***D***: Representative negative control performed in multinucleated cytotrophoblasts at 66 h of culture. Scale bar 50 µm. ***E***, ***F***: IK_Ca_ functional expression. Time course of %^86^Rb efflux over 13 min in multinucleated cytotrophoblasts at 66 h of culture. During the experimental period (indicated by the bar) cells were untreated (control) or treated with DCEBIO, DCEBIO+TRAM-34 (***E***; n = 3 placentas), or with TRAM-34 (***F***; n = 4 placentas). Data are mean ± SE.

IK_Ca_ protein expression was confirmed in mono (Figure 1A) and multinucleated (Figure 1B, C) cytotrophoblasts using immunofluorescent staining with a specific antibody which detects an extracellular site in the pore forming domain (S5–6) of human IK_Ca_ (K_Ca_3.1). IK_Ca_ immunostaining was detected in cells fixed with methanol (intracellular staining; Figures 1A, B) or with PFA (associated with cytotrophoblast surface; Figure 1C).

At 15 h, IK_Ca_ staining (green) was evident in the nucleus (red; nuclear counterstain) of mononucleate cells, but also in the cytoplasm and surface of cell aggregates (Figure 1A). At 66 h, IK_Ca_ was associated to both the cytoplasm (Figure 1B) and cell surface (Figure 1C) of multinucleated cytotrophoblasts. Arrows indicate specific areas were the staining was associated to the cell surface. Figure 1D corresponds to a representative negative control showing that non-specific staining was not observed.

Functional expression of IK_Ca_ was confirmed by measuring ^86^Rb efflux, an indirect assessment of K^+^ permeability, in multinucleated cytotrophoblasts after 66 h of culture. The time course of %^86^Rb efflux/min is plotted in Figure 1E and F. Basal %^86^Rb efflux in control cytotrophoblasts showed a stable steady state over 13 min (Figure 1E; black circles). DCEBIO, an IK_Ca_ activator, caused a marked rapid increase (8.3-fold) in ^86^Rb efflux which was completely blocked by TRAM-34 (85%), an IK_Ca_ inhibitor (Figure 1E). Rate constants, taken as the slopes of the regression lines fitted over the experimental period (10 min), were calculated and for all treatments the data could be fitted by a single exponential (Table 1). The fall in intracellular ^86^Rb (slope) was significantly greater with DCEBIO compared to DCEBIO+TRAM-34 and controls. TRAM-34 had no effect on basal ^86^Rb efflux (Figure 1F). The increase in ^86^Rb efflux with DCEBIO confirms the functional expression of IK_Ca_ in multinucleated cytotrophoblasts.

**Table 1:** Mean rate constants of ^86^Rb efflux in control and treated cytotrophoblasts.

Condition	^86^Rb efflux rate constant (l_n_^86^Rb in cell (t = x)/(t = 0))/min^−1^	r^2^	p value	n
**Control**	−0.015±0.001	0.660	–	8
**Control-100** **µM DCEBIO**	−0.068±0.005*	0.863	<0.0001	3
**Control-100** **µM DCEBIO+10** **µM TRAM-34**	−0.013±0.004	0.284	0.689	3
**Control-10** **µM TRAM-34**	−0,014±0.001**	0.861	0.985	4
**Hyposmotic solution**	−0.032±0.001***	0.933	<0.0001	6
**Hyposmotic solution+100** **nM apamin**	−0.036±0.003†	0.854	0.799	3
**Hyposmotic solution+100** **nM ChTx**	−0.018±0.001†	0.885	<0.0001	3
**Hyposmotic solution+10** **µM TRAM-34**	−0.013±0.001††	0.867	<0.0001	5

Data are mean ± SE, n is the number of placentas. *p* values determined by linear regression;

*compared to corresponding control (−0.011±0.002/min^−1^; r^2^ 0.628) and 100 µM DCEBIO+10 µM TRAM-34 (−0.013±0.004/min^−1^; r^2^ 0.284);

**compared to control (−0.014±0.001/min^−1^; r^2^ 0.823);

***compared to control (−0.013±0.001/min^−1^; r^2^ 0.763);

† compared to hyposmotic solution (−0.035±0.003/min^−1^; r^2^ 0.808);

†† compared to hyposmotic solution (−0.032±0.001/min^−11^; r^2^ 0.925).

### Differentiation of Cytotrophoblasts in Culture

**Figure 2:**
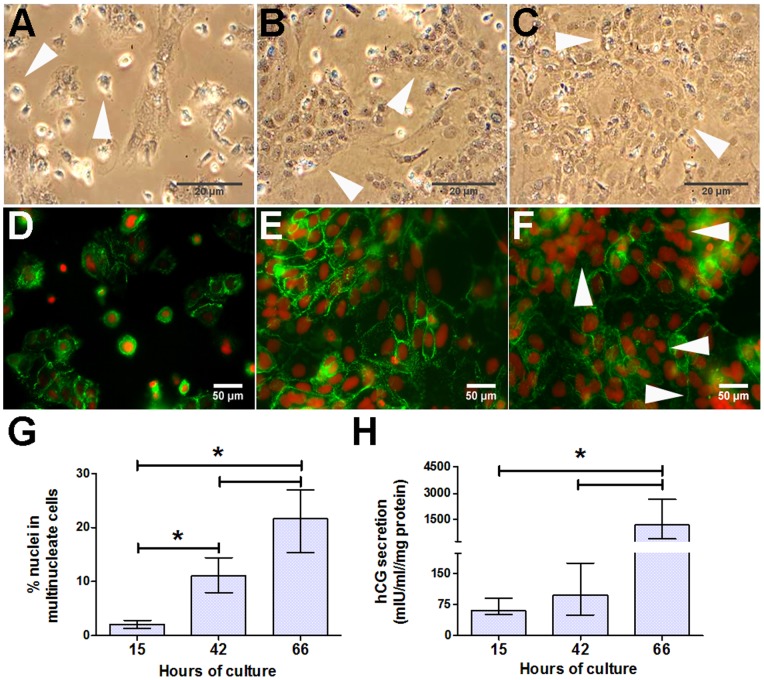
Characterization of placental cytotrophoblasts in culture. Representative phase contrast images of cytotrophoblasts at (***A***) 15 h, (***B***) 42 h and (***C***) 66 h depicting cytotrophoblast morphological differentiation in culture (scale bar 20 µm), together with corresponding immunofluorescent desmoplakin-staining (green) and nuclear counterstain (red; scale bar 50 µm) in ***D***, ***E***, ***F*** respectively. Arrows show different stages of cytotrophoblast differentiation: in (***A***) mononucleate cells, (***B***) multinucleate cell aggregates and (***C***, ***F***) multinucleate syncytial-type cells. ***G***: The % of cytotrophoblast nuclei in multinucleate cells depicting multinucleation and morphological differentiation (n = 15 placentas) at 15, 42 and 66 h in culture. ***H***: Cytotrophoblast β-hCG secretion depicting biochemical differentiation (n = 16 placentas); *p<0.05; Friedman’s test with Dunn’s post hoc test. Data are median ± IQR.

We confirmed previous reports of cytotrophoblast morphological and biochemical differentiation in culture [4], [5], [51]. Figures 2A–C show representative phase contrast images depicting cytotrophoblast morphology. The arrows indicate mononuclear cells at 15 h, aggregates at 42 h and multinucleate cytotrophoblasts at 66 h (in Figures 2A, 2B and 2C respectively). Desmoplakin immunostaining (Figures 2D–F) confirmed this progression of morphological differentiation and was used to calculate the % of nuclei in multinucleate cells (multinucleation) at 15, 42 and 66 h of culture. At 15 h, cytotrophoblasts remained mononuclear (Figure 2D), at 42 h the cells had aggregated (Figure 2E) and at 66 h, cytotrophoblasts had fused to become multinucleated as indicated by the absence of desmoplakin staining (≥3 nuclei in syncytia; Figure 2F). Cytotrophoblast multinucleation increased 12-fold between 15 and 66 h (Figure 2G). This morphological progression was accompanied by biochemical differentiation as indicated by an increase in hCG secretion (Figure 2H). Cytotrophoblast β-hCG secretion increased 20-fold between 15 and 66 h (Figure 2H).

### Effect of IK_Ca_ Modulators on Cytotrophoblast Multinucleation

**Figure 3:**
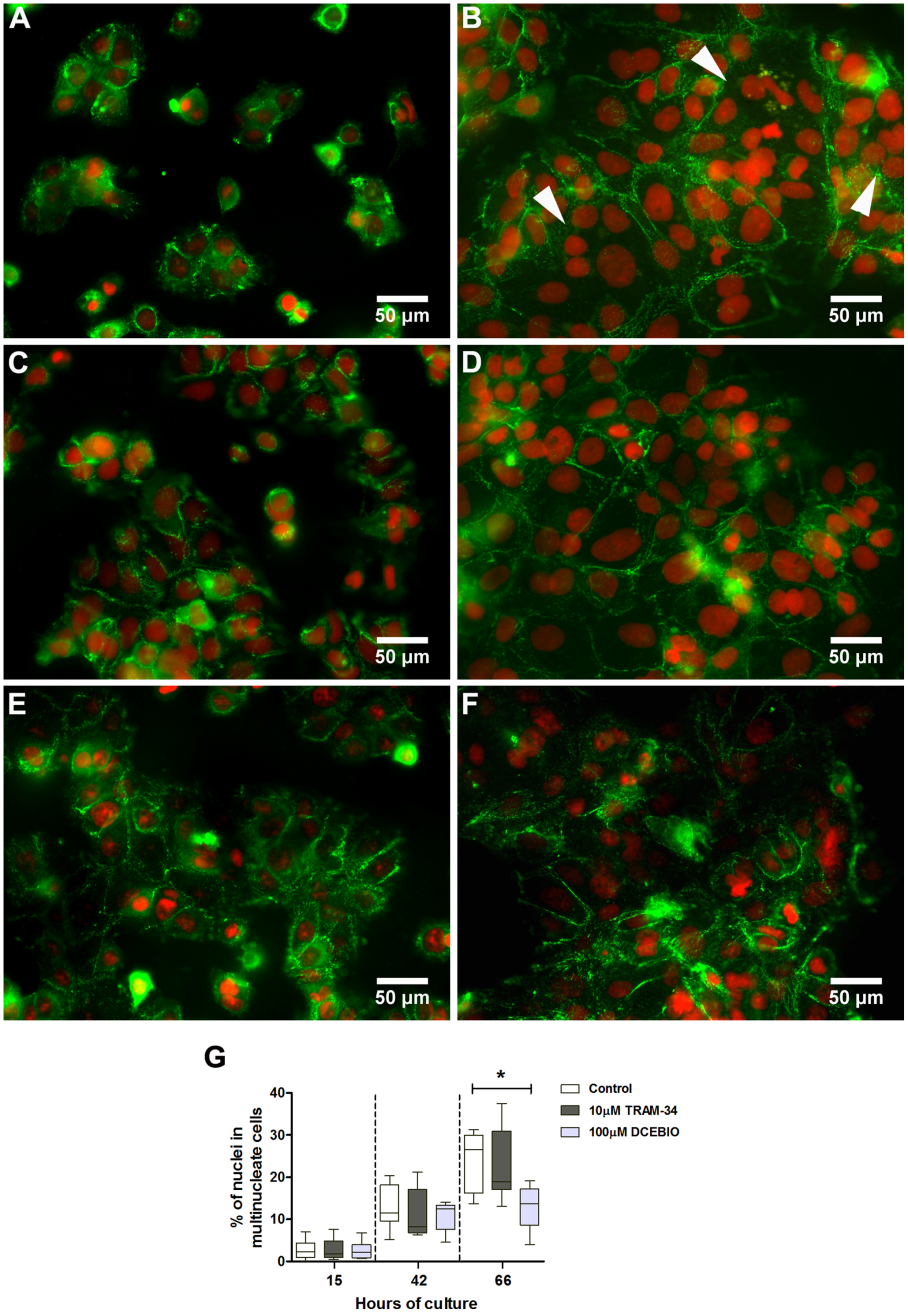
DCEBIO reduces cytotrophoblast multinucleation. Representative dual immunofluorescent staining showing desmoplakin (green) and nuclear counterstain (red) in control untreated (***A***, ***B***), TRAM-34 (***C***, ***D***) or DCEBIO (***E***, ***F***) treated cytotrophoblasts at 15 (***A***, ***C***, ***E***) and 66 h (***B***, ***D***, ***F***) of culture. Arrows in ***B*** indicate multinucleated cytotrophoblasts at 66 h of culture. Scale bar 50 µm. ***G***: The % of cytotrophoblast nuclei in multinucleate cells (multinucleation) at 15, 42 and 66 h of culture (n = 6 placentas); *p<0.05; Friedman’s test with Dunn’s post hoc test. Data are median ± IQR.

Cytotrophoblasts were treated at 3, 15 and 42 h of culture with IK_Ca_ modulators TRAM-34 and DCEBIO and multinucleation (% of nuclei in multinucleate cells) was assessed to determine morphological differentiation. Figures 3A–F show representative images of desmoplakin immunostaining (green) and Pi (red; nuclei) in cytotrophoblasts at 15 (Figure 3A: control untreated, 3C: TRAM-34, 3E: DCEBIO-treated) and 66 h (Figure 3B: control, 3D: TRAM-34, 3F: DCEBIO-treated) of culture. Compared to controls, activation of IK_Ca_ with DCEBIO significantly reduced multinucleation by 42% (median ± IQR: 26.6 *16.2/30.0* compared to 13.8 *8.6/17.2* respectively) at 66 h of culture (Figure 3G). Multinucleation was unaffected by TRAM-34 (Figures 3C, D, G).

### Effect of IK_Ca_ Modulators on Cytotrophoblast hCG Secretion

**Figure 4:**
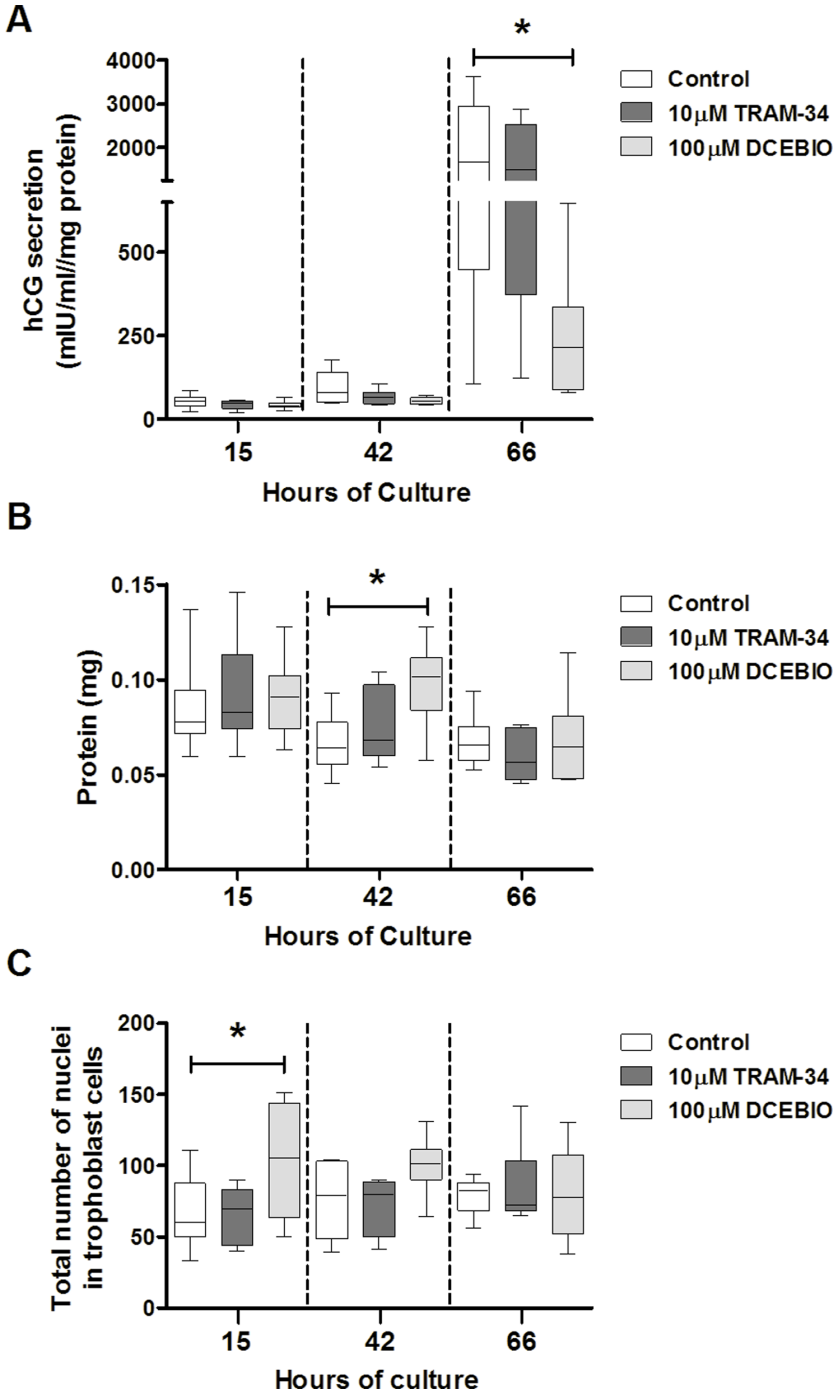
DCEBIO inhibits cytotrophoblast hCG secretion. (***A***) β-hCG secretion, (***B***) cell protein and (***C***) total number of nuclei in cytotrophoblasts at 15, 42 and 66 h of culture in controls and cells treated with TRAM-34 or DCEBIO; n = 6 placentas; *p<0.05; Friedman’s test with Dunn’s post hoc test. Data are median ± IQR.

Compared to controls at 66 h, DCEBIO reduced β-hCG secretion by 80% (19.*5 7.1/19.5*; Figure 4A). This inhibition of differentiation was not associated with a fall in total cell protein (Figure 4B), a proxy measure of cell number, suggesting that DCEBIO did not have a generalized toxic effect. On the contrary, DCEBIO caused a transient increase in cell protein at 42 h (148.8 *134.8/157.1*; Figure 4B). TRAM-34 did not affect cytotrophoblast hCG secretion (Figure 4A) or total cell protein (Figure 4B). In addition, the total number of nuclei was unaffected by the treatment with TRAM-34; however, treatment with DCEBIO caused a transient increase in the total number of nuclei at 15 h of culture (Figure 4C).

### Effect of IK_Ca_ Inhibitor on Swelling-activated K^+^ Efflux from Cytotrophoblasts

A role for IK_Ca_ in regulating syncytiotrophoblast volume was explored using multinucleated cytotrophoblasts. We investigated the participation of IK_Ca_ in syncytiotrophoblast RVD by experimentally exposing cytotrophoblasts to a hyposmotic solution and measuring ^86^Rb efflux as a marker of syncytiotrophoblast K^+^ permeability.

**Figure 5:**
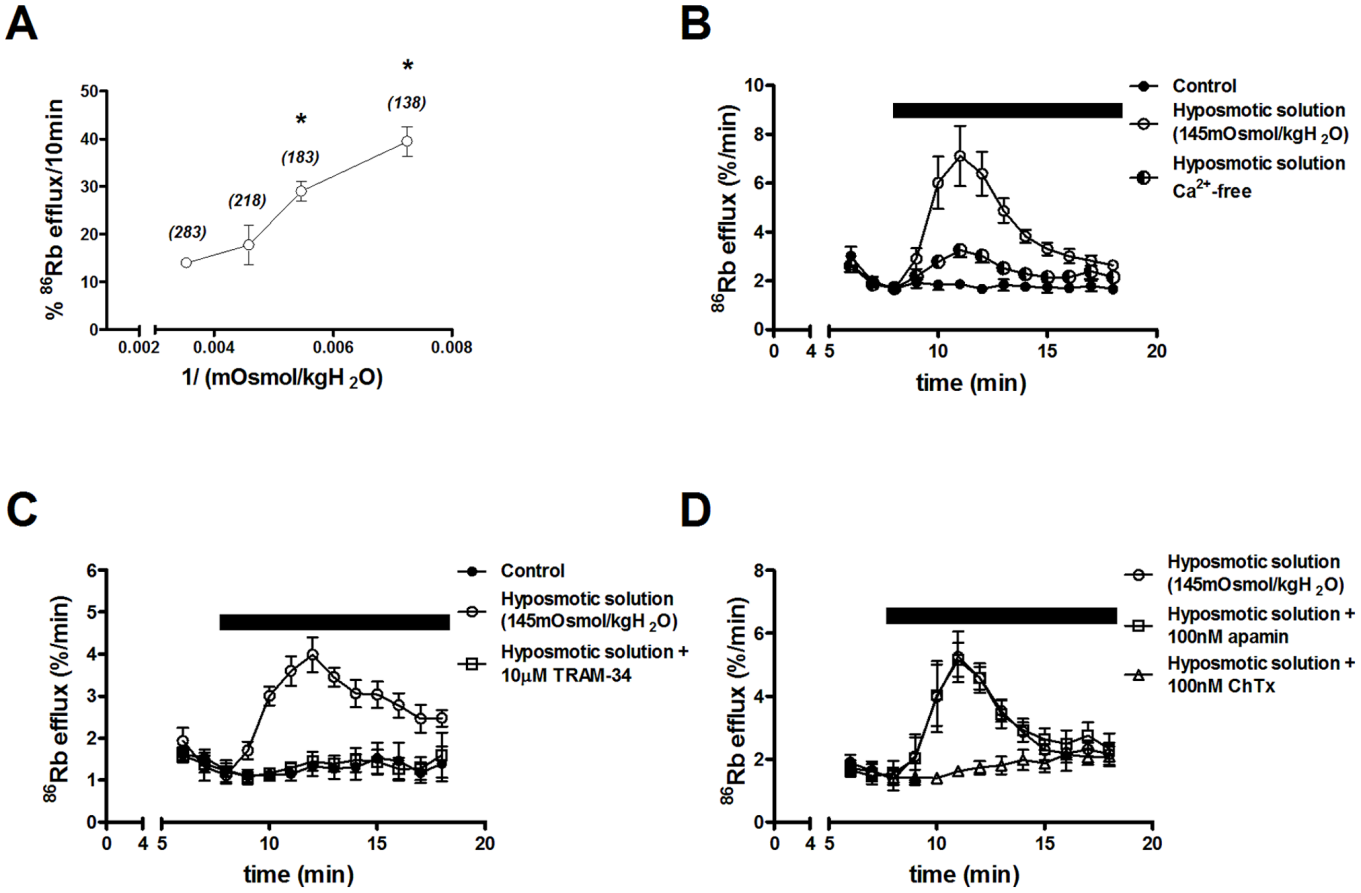
Hypo-osmolality activates IK_Ca_. ***A***: Relationship between ^86^Rb efflux and extracellular fluid osmolality in multinucleated cytotrophoblasts at 66 h culture. The cells were bathed in Tyrode’s buffer with an osmolality of 283 (control), 218, 183 or 138 mOsm/kgH_2_O (shown in italics) for 10 min and the total ^86^Rb efflux over this period was plotted against 1/osmolality. Values are mean ± SE (n = 4–5 placentas); *p<0.001 vs. control at 66 h in culture; ANOVA with Turkey Kramer multicomparison post hoc test. ***B***-***D***: Characterization of swelling-activated ^86^Rb efflux in multinucleated cytotrophoblasts (66 h). Time course of %^86^Rb efflux over 13 min; during the experimental period (indicated by the bar) cells were (***B***) untreated (control) or exposed to hyposmotic solution (145 mOsm/kgH_2_O) or Ca^2+^-free hyposmotic (n = 6 placentas); (***C***) control, hyposmotic solution or hyposmotic solution+TRAM-34 (n = 5 placentas); (***D***) hyposmotic solution, hyposmotic solution+apamin or hyposmotic solution+charybdotoxin (ChTx) (n = 5 placentas). Data are mean ± SE.

Figure 5A shows the relationship between ^86^Rb efflux and extracellular fluid osmolality (ranging from 283–138 mOsm/kgH_2_O). Total ^86^Rb efflux over 10 min (experimental period) was plotted against the reciprocal value for the osmolality of the fluid bathing the cytotrophoblasts after 66 h of culture. A reduction in osmolality to 218 mOsm/kgH_2_O (77% of control), stimulated ^86^Rb efflux compared to control (283 mOsm/kgH_2_O). Reducing extracellular osmolality to 183 and 138 mOsm/kgH_2_O (65 and 49% of control respectively) progressively stimulated ^86^Rb efflux over control. Consequently, the minimum extracellular osmolality required to trigger ^86^Rb efflux from multinucleated cytotrophoblasts is between 77–65% isotonic. Therefore, the remaining experiments were performed using a hyposmotic solution with an osmolality of 145 mOsm/kgH_2_O.

In agreement with previous results in placental villous tissue [57], exposure of multinucleated cytotrophoblasts to a hyposmotic solution markedly increased ^86^Rb efflux (3.8-fold; Figure 5B). The rate constant (Table 1) for ^86^Rb efflux was significantly greater in cytotrophoblasts exposed to the hyposmotic solution than controls. In addition, swelling-activated ^86^Rb efflux was Ca^2+^-dependent, as removal of Ca^2+^ from the hyposmotic solution abolished the activation of ^86^Rb efflux at 66 h of culture (Figure 5B; Table 1).

Figure 5C shows that swelling-activated ^86^Rb efflux was blocked by IK_Ca_ inhibitor, TRAM-34 (85%; Figure 5C). In parallel, rate constant analysis shows a significant difference between hyposmotic solution and TRAM-34, indicating that cytotrophoblast cell swelling activates IK_Ca_ (Table 1). Swelling-activated ^86^Rb efflux is mediated specifically by IK_Ca_ as exposure to the SK_Ca_ inhibitor apamin did not affect the stimulated ^86^Rb efflux. In contrast, exposing cytotrophoblasts to IK_Ca_/BK_Ca_ inhibitor ChTx, almost completely inhibited swelling-activated ^86^Rb efflux (Figure 5D; Table 1), suggesting that the regulation of cytotrophoblast cell volume status is through IK_Ca_.

## Discussion

This study shows that IK_Ca_ protein is expressed by mono- and multinucleate cytotrophoblasts *in vitro*. Multinucleate cells show low inherent IK_Ca_ activity as TRAM-34, an inhibitor of IK_Ca_ did not alter basal ^86^Rb efflux. However, DCEBIO stimulated TRAM-34-sensitive ^86^Rb efflux from multinucleate cells indicating the functional presence of IK_Ca_. Chronic (48 h) application of DCEBIO significantly inhibited both the formation of multinucleate cytotrophoblasts and their secretion of hCG. IK_Ca_, in common with other tissues, may play a role in regulating syncytiotrophoblast volume as experimentally-induced cell swelling activated Ca^2+^-dependent TRAM-34-sensitive ^86^Rb efflux from multinucleated cells.

### IK_Ca_ Expression and Function in Cytotrophoblasts from Term Placentas

Immunofluorescent staining of cytotrophoblasts confirmed the expression of IK_Ca_ protein in mononuclear, aggregated and multinucleated cells. IK_Ca_ staining was associated with the nucleus, cytoplasm and cytotrophoblast cell surface regardless of differentiation stage. Other K^+^ channels, such as K_V_s [58] and K_Ca_s [59], [60] have been localized to the cell nucleus in various cell types; it has been suggested K_Ca_s could control Ca^2+^ release and mobilization within the cell nucleus [59]. In addition, there is evidence of intracellular localization of K_Ca_s which may be associated with different cellular functions in non-placental cell types, e.g. in mitochondria [61], intracellular trafficking [62]. Therefore, the heterogeneous localization of IK_Ca_ could be related to diverse functions that these channels might have in cytotrophoblasts during differentiation.

The functional expression of IK_Ca_ was assessed using ^86^Rb efflux as a tracer of K^+^ efflux. The results indicate that multinucleated cytotrophoblasts express functional IK_Ca_ as exposure to the IK_Ca_ activator DCEBIO, significantly increased ^86^Rb efflux. DCEBIO was specific for IK_Ca_ since this increase in efflux was completely blocked by TRAM-34. However, in a quiescent state IK_Ca_ are inactive as TRAM-34 did not affect basal ^86^Rb efflux. This opens the possibility that different stimuli can activate IK_Ca_ in cytotrophoblasts under physiological/pathophysiological conditions but this remains to be determined.

### Role of IK_Ca_ in Cytotrophoblast Multinucleation

Cytotrophoblasts isolated from term placentas subjected to trypsin-DNAse digestion and Percoll gradient separation are enriched in trophoblast markers and lack contamination from other placental cell types such as, endothelial cells, smooth muscle cells, fibroblasts, or macrophages [4], [5]. After isolation and during the first hours, these cells, which are mitotically inactive, remain mononucleated and secrete small amounts of hCG. After 24 h in culture, they migrate, aggregate and syncytialize by a process of fusion. By 66 h, cytotrophoblasts are predominantly multinucleated syncytial-like cells which secrete high levels of hCG reminiscent of the syncytiotrophoblast *in vivo* [4], [5]. The loss of desmoplakin immunostaining was used to indicate cytotrophoblast fusion and there was, a progressive increase in the formation of multinucleated cytotrophoblasts (≥3 nuclei) after 42 h in culture. Cytotrophoblast differentiation was impaired when IK_Ca_ was activated over 42–66 h. DCEBIO did not alter aggregation but inhibited cytotrophoblast morphological and biochemical differentiation *in vitro* by reducing multinucleation and hCG secretion respectively. These effects were not related to toxicity as total protein was unaffected; indeed, protein levels and the total number of nuclei were higher with DCEBIO at 15–42 h compared to control and this might indicate a transient improved cell viability. Conversely, TRAM-34 treatment did not affect cytotrophoblast syncytialization or hCG secretion. This IK_Ca_ inhibitor was also without effect on ^86^Rb efflux indicating little or no IK_Ca_ activity in syncytiotrophoblast under basal conditions.

In non-placental cell types IK_Ca_ is associated with the regulation of processes that contribute to the maintenance of tissue homeostasis including proliferation [31], , differentiation/fusion [44], [45], cell migration [46]–[48] and apoptosis [49]. Particularly, a ChTx (IK_Ca_ inhibitor)-sensitive K^+^ channel activity is necessary for keratinocyte differentiation [44]. Here we showed that pharmacological activation of IK_Ca_ markedly reduced cytotrophoblast syncytialization implying that IK_Ca_ activation inhibits cytotrophoblast-syncytiotrophoblast fusion. In addition, this evidence suggests IK_Ca_ function could change with cytotrophoblast differentiation and therefore chronically activating these channels could lead to abnormal cytotrophoblast-syncytiotrophoblast fusion and dysregulated turnover. A reduced trophoblast fusion leading to altered syncytiotrophoblast turnover has been proposed in pregnancy complications such as pre-eclampsia as fusogenic proteins are downregulated [22], [24], [27], [28]. However, the specific role of IK_Ca_ in this process, and the cellular signals acting in conjunction to co-ordinate trophoblast fusion, need to be addressed in future.

Despite the well-established role for IK_Ca_s in facilitating cell migration [46]–[48], it is unlikely that they have a similar role in cytotrophoblast migration *in vitro* as cell aggregation, although not assessed quantitatively, did not appear to be affected by openers/inhibitors of IK_Ca_ when applied from 3 h after cell isolation.

### Role of IK_Ca_ in Syncytiotrophoblast Endocrine Secretion

K^+^ channels participate in endocrine secretion [63]–[66] and hCG secretion by placental syncytiotrophoblast is modulated by voltage-gated K^+^ channels (K_V_) [51]. hCG is synthesized and secreted by terminally differentiated trophoblast but the mechanism of secretion is still not fully understood. It is evident that hCG secretion is under autocrine/paracrine regulation by hCG which itself promotes cytotrophoblast cell differentiation and further hCG secretion [67]. K_V_s regulate the secretory process rather than hormone production [51]. Here we showed that the chronic activation of IK_Ca_ significantly reduced hCG secretion by cytotrophoblasts, suggesting that IK_Ca_ could inhibit the mechanism of hCG secretion. However, there is little evidence to link IK_Ca_ function with endocrine secretion, and IK_Ca_ action on hormone secretion is restricted to the central nervous system [68]. We speculate that the primary effect of IK_Ca_ is to inhibit cytotrophoblast cell fusion/terminal differentiation and, as a result, hCG production and secretion is reduced.

### Role of IK_Ca_ in Syncytiotrophoblast Volume Regulation

In many cell types, restoration of cell volume in the presence of a hyposmotic stimulus (RVD) is mediated by K^+^ channels, including IK_Ca_, in conjunction with swelling-activated anion channels [39]. In the current study, exposing multinucleated cytotrophoblasts to a hyposmotic solution increased ^86^Rb efflux ∼3.8-fold and this activated efflux was dependent on extracellular Ca^2+^, blocked (>80%) by the IK_Ca_ inhibitors TRAM-34 and ChTx but was unaffected by the SK_Ca_ inhibitor apamin. These data implicate IK_Ca_ in cytotrophoblast RVD. Lowering extracellular osmolality also stimulated ^86^Rb efflux from placental villous tissue [57] and caused a Ba^2+^-sensitive hyperpolarization of the syncytiotrophoblast microvillous membrane [69]; however, the identities of the K^+^ channels underlying the resting conductance, or the change with cell swelling, remain unknown.

Exposing cells to a hyposmotic solution is an experimental maneuver often used to mimic the cell swelling which takes place secondary to a rise in intracellular osmolality as can occur following nutrient uptake [39], [69], [70]. In this case, activation of K^+^ channels is a homeostatic process to promote water loss to restore the concentration of cytoplasmic constituents and to shrink cells back to their original size. On the other hand, in the absence of hyposmotic swelling, the activation of K^+^ channels to promote water loss effects a cell volume change, and/or fall in intracellular K^+^, that is essential for a variety of processes that maintain tissue homeostasis such as cell proliferation, migration, differentiation/fusion and cell death [71]. It is possible that dynamic changes in cell volume are required for normal cytotrophoblast fusion and that, in the current study, chronically activating IK_Ca_ channels induced an inappropriate change in cell volume which inhibited fusion. Cytotrophoblast fusion may be altered by promoting IK_Ca_ activity and consequently inducing water loss which alters the concentration of cytoplasmic factors that regulate fusion. These proposals need to be investigated in future, and in particular elucidate whether the primary effect of activation of IK_Ca_ is on fusion.

## Conclusions

The primary stimuli for IK_Ca_ activation is an elevation in [Ca^2+^]_i_ and therefore factors that increase [Ca^2+^]_i_ will activate cytotrophoblast IK_Ca_. To date there are relatively few studies of the regulation of [Ca^2+^]_i_ in syncytiotrophoblast; however, preliminary evidence indicates that hyposmotic swelling increases [Ca^2+^]_i_ in multinucleated cytotrophoblasts, predominantly by entry from extracellular fluid [72].

Consequently, activation of IK_Ca_ could regulate syncytiotrophoblast volume, which may change dynamically following solute uptake and/or cytotrophoblast cell fusion, an essential homeostatic mechanism to maintain nutrient transport and endocrine function respectively. In addition, we have previously shown that cytotrophoblast [Ca^2+^]_i_ is elevated following activation of purinergic receptors, including P2X4, by extracellular nucleotides and that this promotes ^86^Rb efflux which is inhibited by ChTx, implicating activation of IK_Ca_. These findings might be of relevance to the etiology of pre-eclampsia, a disease of pregnancy characterized by abnormal cytotrophoblast fusion and renewal of syncytiotrophoblast. Indeed, the expression of P2X4 by the placenta is elevated in pre-eclampsia compared to normal pregnancy [73]. It is also proposed that hypoxia/elevated reactive oxygen species release nucleotides from the trophoblast in pre-eclampsia to elevate local concentrations in the extracellular fluid [73], [74]. As a result, increased activation of P2X4 would elevate [Ca^2+^]_i_ and activate IK_Ca_s. The inappropriate activation of IK_Ca_ could compromise cell volume homeostasis, and impact on cytotrophoblast cell fusion and syncytiotrophoblast renewal, endocrine function and nutrient transport in pre-eclampsia.

## References

[1] Huppertz B, Gauster M (2011) Trophoblast Fusion. In: Dittmar T, Zänker KS, editors. Cell Fusion in Health and Disease: Springer Netherlands. pp. 81–95.

[2] Longtine MS, Chen B, Odibo AO, Zhong Y, Nelson DM (2012) Caspase-mediated apoptosis of trophoblasts in term human placental villi is restricted to cytotrophoblasts and absent from the multinucleated syncytiotrophoblast. Reproduction 143: 107–121. 22046053 10.1530/REP-11-0340 PMC3631347

[3] Longtine MS, Barton A, Chen B, Nelson DM (2012) Live-cell imaging shows apoptosis initiates locally and propagates as a wave throughout syncytiotrophoblasts in primary cultures of human placental villous trophoblasts. Placenta 33: 971–976. 23102999 10.1016/j.placenta.2012.09.013 PMC3505883

[4] Greenwood SL, Brown PD, Edwards D, Sibley CP (1993) Patch clamp studies of human placental cytotrophoblast cells in culture. Placenta 14: 53–68.

[5] Kliman HJ, Nestler JE, Sermasi E, Sanger JM, Strauss JF 3rd (1986) Purification, characterization, and in vitro differentiation of cytotrophoblasts from human term placentae. Endocrinology 118: 1567–1582. 3512258 10.1210/endo-118-4-1567

[6] Douglas GC, King BF (1990) Differentiation of human trophoblast cells in vitro as revealed by immunocytochemical staining of desmoplakin and nuclei. J Cell Sci 96 (Pt 1): 131–141. 10.1242/jcs.96.1.131 2165075

[7] Douglas GC, King BF (1989) Isolation of pure villous cytotrophoblast from term human placenta using immunomagnetic microspheres. J Immunol Methods 119: 259–268. 2470826 10.1016/0022-1759(89)90405-5

[8] Yang M, Lei ZM, Rao Ch V (2003) The central role of human chorionic gonadotropin in the formation of human placental syncytium. Endocrinology 144: 1108–1120. 12586787 10.1210/en.2002-220922

[9] Cronier L, Bastide B, Herve JC, Deleze J, Malassine A (1994) Gap junctional communication during human trophoblast differentiation: influence of human chorionic gonadotropin. Endocrinology 135: 402–408. 8013377 10.1210/endo.135.1.8013377

[10] Lim KH, Zhou Y, Janatpour M, McMaster M, Bass K, et al (1997) Human cytotrophoblast differentiation/invasion is abnormal in pre-eclampsia. Am J Pathol 151: 1809–1818. 9403732 PMC1858365

[11] Crocker IP, Tansinda DM, Baker PN (2004) Altered cell kinetics in cultured placental villous explants in pregnancies complicated by pre-eclampsia and intrauterine growth restriction. J Pathol 204: 11–18. 15307133 10.1002/path.1610

[12] Huppertz B, Kaufmann P, Kingdom J (2002) Trophoblast turnover in health and disease. Fetal Matern Med Rev 13: 103–118.

[13] Smith SC, Baker PN, Symonds EM (1997) Increased placental apoptosis in intrauterine growth restriction. Am J Obstet Gynecol 177: 1395–1401. 9423741 10.1016/s0002-9378(97)70081-4

[14] Higgins L, Mills TA, Greenwood SL, Cowley EJ, Sibley CP, et al (2012) Maternal obesity and its effect on placental cell turnover. J Matern Fetal Neonatal Med 26: 783–788. 10.3109/14767058.2012.760539 23270521

[15] Arnholdt H, Meisel F, Fandrey K, Lohrs U (1991) Proliferation of villous trophoblast of the human placenta in normal and abnormal pregnancies. Virchows Arch B Cell Pathol Incl Mol Pathol 60: 365–372. 1683053 10.1007/BF02899568

[16] Brown LM, Lacey HA, Baker PN, Crocker IP (2005) E-cadherin in the assessment of aberrant placental cytotrophoblast turnover in pregnancies complicated by pre-eclampsia. Histochem Cell Biol 124: 499–506. 16142450 10.1007/s00418-005-0051-7

[17] Allaire AD, Ballenger KA, Wells SR, McMahon MJ, Lessey BA (2000) Placental apoptosis in preeclampsia. Obstet Gynecol 96: 271–276. 10908776 10.1016/s0029-7844(00)00895-4

[18] Ishihara N, Matsuo H, Murakoshi H, Laoag-Fernandez JB, Samoto T, et al (2002) Increased apoptosis in the syncytiotrophoblast in human term placentas complicated by either preeclampsia or intrauterine growth retardation. Am J Obstet Gynecol 186: 158–166. 11810103 10.1067/mob.2002.119176

[19] Leung DN, Smith SC, To KF, Sahota DS, Baker PN (2001) Increased placental apoptosis in pregnancies complicated by preeclampsia. Am J Obstet Gynecol 184: 1249–1250. 11349196 10.1067/mob.2001.112906

[20] Correa RR, Gilio DB, Cavellani CL, Paschoini MC, Oliveira FA, et al (2008) Placental morphometrical and histopathology changes in the different clinical presentations of hypertensive syndromes in pregnancy. Arch Gynecol Obstet 277: 201–206. 17786461 10.1007/s00404-007-0452-z

[21] Huppertz B, Kingdom JC (2004) Apoptosis in the trophoblast-role of apoptosis in placental morphogenesis. J Soc Gynecol Investig 11: 353–362. 10.1016/j.jsgi.2004.06.002 15350247

[22] Langbein M, Strick R, Strissel PL, Vogt N, Parsch H, et al (2008) Impaired cytotrophoblast cell-cell fusion is associated with reduced Syncytin and increased apoptosis in patients with placental dysfunction. Mol Reprod Dev 75: 175–183. 17546632 10.1002/mrd.20729

[23] Mi S, Lee X, Li X, Veldman GM, Finnerty H, et al (2000) Syncytin is a captive retroviral envelope protein involved in human placental morphogenesis. Nature 403: 785–789. 10693809 10.1038/35001608

[24] Lee X, Keith JC Jr, Stumm N, Moutsatsos I, McCoy JM, et al (2001) Downregulation of placental syncytin expression and abnormal protein localization in pre-eclampsia. Placenta 22: 808–812. 11718567 10.1053/plac.2001.0722

[25] Frendo JL, Olivier D, Cheynet V, Blond JL, Bouton O, et al (2003) Direct involvement of HERV-W Env glycoprotein in human trophoblast cell fusion and differentiation. Mol Cell Biol 23: 3566–3574. 12724415 10.1128/MCB.23.10.3566-3574.2003 PMC164757

[26] Vargas A, Moreau J, Landry S, LeBellego F, Toufaily C, et al (2009) Syncytin-2 plays an important role in the fusion of human trophoblast cells. J Mol Biol 392: 301–318. 19616006 10.1016/j.jmb.2009.07.025

[27] Keith JC, Jr., Pijnenborg R, Van Assche FA (2002) Placental syncytin expression in normal and preeclamptic pregnancies. Am J Obstet Gynecol 187: 1122–1123; author reply 1123–1124. 10.1067/mob.2002.128512 12389018

[28] Vargas A, Toufaily C, LeBellego F, Rassart E, Lafond J, et al (2011) Reduced expression of both syncytin 1 and syncytin 2 correlates with severity of preeclampsia. Reprod Sci 18: 1085–1091. 21493955 10.1177/1933719111404608

[29] Mayhew TM (2009) A stereological perspective on placental morphology in normal and complicated pregnancies. J Anat 215: 77–90. 19141109 10.1111/j.1469-7580.2008.00994.x PMC2714641

[30] Mayhew TM, Manwani R, Ohadike C, Wijesekara J, Baker PN (2007) The placenta in pre-eclampsia and intrauterine growth restriction: studies on exchange surface areas, diffusion distances and villous membrane diffusive conductances. Placenta 28: 233–238. 16635527 10.1016/j.placenta.2006.02.011

[31] Neylon CB, Lang RJ, Fu Y, Bobik A, Reinhart PH (1999) Molecular cloning and characterization of the intermediate-conductance Ca(2+)-activated K(+) channel in vascular smooth muscle: relationship between K(Ca) channel diversity and smooth muscle cell function. Circ Res 85: e33–43. 10532960 10.1161/01.res.85.9.e33

[32] Wei AD, Gutman GA, Aldrich R, Chandy KG, Grissmer S, et al (2005) International Union of Pharmacology. LII. Nomenclature and Molecular Relationships of Calcium-Activated Potassium Channels. Pharmacol Rev 57: 463–472. 16382103 10.1124/pr.57.4.9

[33] Jensen BS, Strobak D, Christophersen P, Jorgensen TD, Hansen C, et al (1998) Characterization of the cloned human intermediate-conductance Ca2+-activated K+ channel. Am J Physiol Cell Physiol 275: C848–856. 10.1152/ajpcell.1998.275.3.C848 9730970

[34] Weskamp M, Seidl W, Grissmer S (2000) Characterization of the increase in [Ca(2+)](i) during hypotonic shock and the involvement of Ca(2+)-activated K(+) channels in the regulatory volume decrease in human osteoblast-like cells. J Membr Biol 178: 11–20. 11058683 10.1007/s002320010010

[35] Wang J, Morishima S, Okada Y (2003) IK channels are involved in the regulatory volume decrease in human epithelial cells. Am J Physiol Cell Physiol 284: C77–84. 12388088 10.1152/ajpcell.00132.2002

[36] Barfod ET, Moore AL, Roe MW, Lidofsky SD (2007) Ca2+-activated IK1 Channels Associate with Lipid Rafts upon Cell Swelling and Mediate Volume Recovery. J Biol Chem 282: 8984–8993. 17264085 10.1074/jbc.M607730200

[37] Vázquez E, Nobles M, Valverde MA (2001) Defective regulatory volume decrease in human cystic fibrosis tracheal cells because of altered regulation of intermediate conductance Ca2+-dependent potassium channels. Proc Natl Acad Sci U S A 98: 5329–5334. 11309505 10.1073/pnas.091096498 PMC33209

[38] Sand P, Anger A, Rydqvist B (2004) Hypotonic stress activates an intermediate conductance K+ channel in human colonic crypt cells. Acta Physiol Scand 182: 361–368. 15569097 10.1111/j.1365-201X.2004.01366.x

[39] Hoffmann EK, Lambert IH, Pedersen SF (2009) Physiology of cell volume regulation in vertebrates. Physiol Rev 89: 193–277. 19126758 10.1152/physrev.00037.2007

[40] Lang F, Busch GL, Ritter M, Volkl H, Waldegger S, et al (1998) Functional significance of cell volume regulatory mechanisms. Physiol Rev 78: 247–306. 9457175 10.1152/physrev.1998.78.1.247

[41] Tao R, Lau CP, Tse HF, Li GR (2008) Regulation of cell proliferation by intermediate-conductance Ca2+-activated potassium and volume-sensitive chloride channels in mouse mesenchymal stem cells. Am J Physiol Cell Physiol 295: C1409–1416. 18815226 10.1152/ajpcell.00268.2008 PMC3817252

[42] Cheong A, Bingham AJ, Li J, Kumar B, Sukumar P, et al (2005) Downregulated REST transcription factor is a switch enabling critical potassium channel expression and cell proliferation. Mol Cell 20: 45–52. 16209944 10.1016/j.molcel.2005.08.030

[43] Millership JE, Devor DC, Hamilton KL, Balut CM, Bruce JI, et al (2011) Calcium-activated K+ channels increase cell proliferation independent of K+ conductance. Am J Physiol Cell Physiol 300: C792–802. 21123738 10.1152/ajpcell.00274.2010 PMC3074627

[44] Mauro T, Dixon DB, Komuves L, Hanley K, Pappone PA (1997) Keratinocyte K+ channels mediate Ca2+-induced differentiation. J Invest Dermatol 108: 864–870. 9182812 10.1111/1523-1747.ep12292585

[45] Fioretti B, Pietrangelo T, Catacuzzeno L, Franciolini F (2005) Intermediate-conductance Ca2+-activated K+ channel is expressed in C2C12 myoblasts and is downregulated during myogenesis. Am J Physiol Cell Physiol 289: C89–C96. 15743891 10.1152/ajpcell.00369.2004

[46] Schwab A, Fabian A, Hanley PJ, Stock C (2012) Role of ion channels and transporters in cell migration. Physiol Rev 92: 1865–1913. 23073633 10.1152/physrev.00018.2011

[47] Schwab A, Wulf A, Schulz C, Kessler W, Nechyporuk-Zloy V, et al (2006) Subcellular distribution of calcium-sensitive potassium channels (IK1) in migrating cells. J Cell Physiol 206: 86–94. 15965951 10.1002/jcp.20434

[48] Schwab A, Gabriel K, Finsterwalder F, Folprecht G, Greger R, et al (1995) Polarized ion transport during migration of transformed Madin-Darby canine kidney cells. Pflugers Arch 430: 802–807. 7478936 10.1007/BF00386179

[49] Lang PA, Kaiser S, Myssina S, Wieder T, Lang F, et al (2003) Role of Ca2+-activated K+ channels in human erythrocyte apoptosis. Am J Physiol Cell Physiol 285: C1553–1560. 14600080 10.1152/ajpcell.00186.2003

[50] Grunnet M, MacAulay N, Jorgensen NK, Jensen S, Olesen SP, et al (2002) Regulation of cloned, Ca2+-activated K+ channels by cell volume changes. Pflugers Arch 444: 167–177. 11976929 10.1007/s00424-002-0782-4

[51] Williams JL, Fyfe GK, Sibley CP, Baker PN, Greenwood SL (2008) K+ channel inhibition modulates the biochemical and morphological differentiation of human placental cytotrophoblast cells in vitro. Am J Physiol Regul Integr Comp Physiol 295: R1204–1213. 18703414 10.1152/ajpregu.00193.2008

[52] Clarson LH, Greenwood SL, Mylona P, Sibley CP (2001) Inwardly rectifying K(+) current and differentiation of human placental cytotrophoblast cells in culture. Placenta 22: 328–336. 11286569 10.1053/plac.2000.0622

[53] Clarson LH, Roberts VH, Greenwood SL, Elliott AC (2002) ATP-stimulated Ca(2+)-activated K(+) efflux pathway and differentiation of human placental cytotrophoblast cells. Am J Physiol Regul Integr Comp Physiol 282: R1077–1085. 11893612 10.1152/ajpregu.00564.2001

[54] Desforges M, Parsons L, Westwood M, Sibley CP, Greenwood SL (2013) Taurine transport in human placental trophoblast is important for regulation of cell differentiation and survival. Cell Death Dis 4: e559. 23519128 10.1038/cddis.2013.81 PMC3618382

[55] Johnstone ED, Sibley CP, Lowen B, Guilbert LJ (2005) Epidermal growth factor stimulation of trophoblast differentiation requires MAPK11/14 (p38 MAP kinase) activation. Biol Reprod 73: 1282–1288. 16120828 10.1095/biolreprod.105.044206

[56] Robertson EG, Cheyne GA (1972) Plasma biochemistry in relation to oedema of pregnancy. J Obstet Gynaecol Br Commonw 79: 769–776. 4651284 10.1111/j.1471-0528.1972.tb12918.x

[57] Siman CM, Sibley CP, Jones CJ, Turner MA, Greenwood SL (2001) The functional regeneration of syncytiotrophoblast in cultured explants of term placenta. Am J Physiol Regul Integr Comp Physiol 280: R1116–1122. 11247834 10.1152/ajpregu.2001.280.4.R1116

[58] Chen Y, Sánchez A, Rubio ME, Kohl T, Pardo LA, et al (2011) Functional KV10.1 Channels Localize to the Inner Nuclear Membrane. PLoS One 6: e19257. 21559285 10.1371/journal.pone.0019257 PMC3086910

[59] Maruyama Y, Shimada H, Taniguchi J (1995) Ca(2+)-activated K(+)-channels in the nuclear envelope isolated from single pancreatic acinar cells. Pflugers Arch 430: 148–150. 7667076 10.1007/BF00373851

[60] Miller MJ, Rauer H, Tomita H, Rauer H, Gargus JJ, et al (2001) Nuclear Localization and Dominant-negative Suppression by a Mutant SKCa3 N-terminal Channel Fragment Identified in a Patient with Schizophrenia. J Biol Chem 276: 27753–27756. 11395478 10.1074/jbc.C100221200

[61] Redel A, Lange M, Jazbutyte V, Lotz C, Smul TM, et al (2008) Activation of Mitochondrial Large-Conductance Calcium-Activated K+ Channels via Protein Kinase A Mediates Desflurane-Induced Preconditioning. Anesth Analg 106: 384–391. 18227289 10.1213/ane.0b013e318160650f

[62] Corrêa SAL, Müller J, Collingridge GL, Marrion NV (2009) Rapid endocytosis provides restricted somatic expression of a K+ channel in central neurons. J Cell Sci 122: 4186–4194. 19861491 10.1242/jcs.058420 PMC2776504

[63] Jacobson DA, Kuznetsov A, Lopez JP, Kash S, Ammala CE, et al (2007) Kv2.1 ablation alters glucose-induced islet electrical activity, enhancing insulin secretion. Cell Metab 6: 229–235. 17767909 10.1016/j.cmet.2007.07.010 PMC2699758

[64] Ashcroft FM, Gribble FM (1999) ATP-sensitive K+ channels and insulin secretion: their role in health and disease. Diabetologia 42: 903–919. 10491749 10.1007/s001250051247

[65] Leung YM, Kwan EP, Ng B, Kang Y, Gaisano HY (2007) SNAREing voltage-gated K+ and ATP-sensitive K+ channels: tuning beta-cell excitability with syntaxin-1A and other exocytotic proteins. Endocr Rev 28: 653–663. 17878408 10.1210/er.2007-0010

[66] Li X, Herrington J, Petrov A, Ge L, Eiermann G, et al (2013) The Role of Voltage-Gated Potassium Channels Kv2.1 and Kv2.2 in the Regulation of Insulin and Somatostatin Release from Pancreatic Islets. J Pharmacol Exp Ther 344: 407–416. 23161216 10.1124/jpet.112.199083

[67] Shi QJ, Lei ZM, Rao CV, Lin J (1993) Novel role of human chorionic gonadotropin in differentiation of human cytotrophoblasts. Endocrinology 132: 1387–1395. 7679981 10.1210/endo.132.3.7679981

[68] Liang Z, Chen L, McClafferty H, Lukowski R, MacGregor D, et al (2011) Control of hypothalamic–pituitary–adrenal stress axis activity by the intermediate conductance calcium-activated potassium channel, SK4. J Physiol 589: 5965–5986. 22041182 10.1113/jphysiol.2011.219378 PMC3286679

[69] Birdsey TJ, Boyd RD, Sibley CP, Greenwood SL (1999) Effect of hyposmotic challenge on microvillous membrane potential in isolated human placental villi. Am J Physiol 276: R1479–1488. 10233042 10.1152/ajpregu.1999.276.5.R1479

[70] Gow IF, Thomson J, Davidson J, Shennan DB (2005) The effect of a hyposmotic shock and purinergic agonists on K+(Rb+) efflux from cultured human breast cancer cells. Biochim Biophys Acta 1712: 52–61. 15890311 10.1016/j.bbamem.2005.04.002

[71] Okada Y, Maeno E, Shimizu T, Dezaki K, Wang J, et al (2001) Receptor-mediated control of regulatory volume decrease (RVD) and apoptotic volume decrease (AVD). J Physiol 532: 3–16. 11283221 10.1111/j.1469-7793.2001.0003g.x PMC2278524

[72] van de Put FHMM, Greenwood SL, Sibley CP (1996) Effect of hyposmotic cell swelling on [Ca2+]i in human placental cytotrophoblast cells in culture. Placenta 17: A15.

[73] Roberts VH, Webster RP, Brockman DE, Pitzer BA, Myatt L (2007) Post-Translational Modifications of the P2X(4) purinergic receptor subtype in the human placenta are altered in preeclampsia. Placenta 28: 270–277. 16793133 10.1016/j.placenta.2006.04.008

[74] Bakker WW, Donker RB, Timmer A, van Pampus MG, van Son WJ, et al (2007) Plasma Hemopexin Activity in Pregnancy and Preeclampsia. Hypertens Pregnancy 26: 227–239. 17469012 10.1080/10641950701274896

